# A Case for Counsel

**Published:** 1888-02

**Authors:** M. R. Jamison

**Affiliations:** Connellsville, Pa.


### A CASE FOR COUNSEL.

Miss A., age twenty-eight, a lady of excellent moral character and good family history, except consumption on the father's side, her only brother dying of this disease. Patient has been subject all her life to very violent headaches or neuralgias; when only a child, was ill for two days at a time with violent pains in the head. Character and location of these pains she does not remember. When in her teens was subject to violent dysmenorrhœa; has had typhoid fever. Two years ago had acute inflammatory rheumatism. For five years has had post-nasal and laryngeal catarrh. Several polypi have been removed from nares, and the parts cauterized. Two years ago the walls of the pharynx were studded with small ulcers. She has taken enough drugs to float the Great Eastern, and yet lives. Eight months ago I was called to relieve this lady of a very violent neuralgia, which I was more successful in doing than my allopathic brother, and the case was soon put under my care. I found my patient with the following symptoms, which have characterized her case all along:

Violent pain in left eyeball; the eye feels as if it would burst, or as if a knife was plunged into it; pains shot from the eye to vertex; a very violent *drawing* pain on the vertex; the eye burns like fire; watering of the eye; pains radiate from eye in all directions; eyeball and adjacent parts exceedingly sensitive to touch; pains in occiput and cervical region; violent pain with intense burning in left nostril; pain at the bridge of the nose; left side of nose very sensitive to touch; complete stoppage of the nose; can't breathe through nares; tip of the nose very red; very nervous, throwing arms about; sometimes fluttering of the heart; alternately chilly and hot; hands and feet cold. If patient is not soon relieved the suffering becomes so great she screams with pain; the face, lips, and tongue tingle; the hands cramp, the fingers spreading; the pulse so feeble can scarcely be counted; twitching of the muscles of forearm, and my patient becomes unconscious. In a few minutes consciousness returns and the pains slowly subside. The attacks come without any apparent cause, though excitement has been known to produce attack. In some attacks the remedy soon gives relief; the next time the remedy is powerless to relieve. Acon., Bell., Ars., Cup. ars., Cup. met., Act., Rac., and Spig. have been given, but none of them give relief nearly so quickly as I usually receive from a well-chosen remedy in neuralgia. Many times my patient must suffer from ten to fifteen hours before relief can be had. There ought to be a remedy that would relieve my patient in two or three hours. What is the remedy?

In the interval of these attacks, the patient complains more or less of the following symptoms: Dull pain and aching in occiput and cervical region; muscles in this region are sore; dull pain and aching between the eyes; great burning in posterior nares and throat; stinging and aching in throat; posterior nares feel raw and sore; thick, yellow, sometimes stringy, discharges from nose. Walls of the larynx red, dry, ragged, and pitted. Aphonia appearing without catarrhal symptoms lasting from a few days to eighteen weeks, suddenly disappearing.

When not troubled with aphonia, the voice is clear and musical, and the patient sings in the choir every Sabbath. Attacks of dyspnœa; wants the doors and windows open and to be fanned. For many days will have slight difficulty in breathing. Burning and stinging in apex of left lung, and a sense of great weight there. Sight of left eye deficient; objects look dim; letters run together. Swelling of the body, neck and face, and upper and lower limbs. Knees are very much swollen. Hands suddenly swell, must remove gloves in church. The arms ache and feel heavy as lead. Arms and hands feel as if gone to sleep; sleepy, but cannot sleep; slight noises keep her awake; suddenly starts on falling asleep; awakens in sleep with a start. If she does sleep soundly, feels utterly prostrated on awaking, almost powerless to move. Menses regular, but too scanty, last but two days; very dark and clotted, no pain. In early menstrual life, the menses very irregular; six to eight weeks intervals. The limbs ache and the muscles are sore; fidgety feet. For these symptoms I have given Sul., Act-rac., Kali-bich., and Ars., with indifferent results. My patient is but little better to-day than when I began the treatment. Is the lack of success in the selection of the remedy? Is the seat of the difficulty in the base of the brain, or where?

There is one spot in the post nasal cavity which if touched will produce instantly this drawing pain in the vertex.

Perhaps the nasal catarrh has something to do with this violent neuralgia. M. R. Jamison, M. D.

Connellsville, Pa.

